# Prevalence and intensity of neglected tropical diseases (schistosomiasis and soil-transmitted helminths) amongst rural female pupils in Ugu district, KwaZulu-Natal, South Africa

**DOI:** 10.4102/sajid.v35i1.123

**Published:** 2020-06-03

**Authors:** Siphosenkosi G. Zulu, Eyrun F. Kjetland, Svein G. Gundersen, Myra Taylor

**Affiliations:** 1 Discipline of Public Health Medicine, Department of Medical Microbiology, Nelson R Mandela School of Medicine, College of Health Sciences, University of KwaZulu-Natal, Durban, South Africa; 2 Norwegian Centre for Imported and Tropical Diseases, Department of Infectious Diseases, Oslo University Hospital Ullevaal, Oslo, Norway; 3 Oslo University Hospital, Oslo, Norway; 4 Institute of Clinical Medicine, University of Oslo, Oslo, Norway; 5 Research Unit, Sorlandet Hospital, Kristiansand, Norway; 6 Department of Global Development and Planning, University of Agder, Kristiansand, Norway; 7 Nelson R Mandela School of Medicine, College of Health Sciences, University of KwaZulu-Natal, Durban, South Africa

## Abstract

**Background:**

Inadequate water supply and sanitation adversely affects the health and socio-economic development of communities and places them at risk of contracting schistosomiasis and soil-transmitted helminths (STHs). The aim of this study was to quantify the prevalence and intensity of schistosomiasis (bilharzia) and STHs amongst female school-going pupils in Ugu district.

**Methods:**

A descriptive cross-sectional study was conducted in Ugu district amongst primary school pupils from 18 randomly selected schools in 2010. A structured questionnaire was used to collect information on the history and knowledge of bilharzia of 1057 pupils. One stool and 3 consecutive days of urine samples were collected per participant and screened for helminth ova. Findings were compared with those reported by the parasite control programme, which collected data in the same area in 1998.

**Results:**

The prevalence of *Ascaris lumbricoides* and *Trichuris trichiura* was 25% and 26%, respectively, and their corresponding mean intensities of infection were 21 and 26 eggs per gram. The prevalence of *Schistosoma haematobium* was 32%, and its mean intensity of infection was 52 eggs per 10 mL urine. Of the pupils, 60% knew about schistosomiasis, 9% reported red urine in the past week and 22% had had dysuria before. Although the prevalence of ascariasis and trichuriasis had decreased since 1998 (62% and 59%, respectively), the prevalence of schistosomiasis had increased to 32% (*p* < 0.05).

**Conclusion:**

Female pupils in rural schools remain at risk. A mass treatment campaign, increased public awareness and improved sanitation are required to reduce these infections and sustain a reduction of STHs and schistosomiasis.

**Keywords:**

prevalence; intensity; schistosomiasis; soil-transmitted helminths; *Ascaris lumbricoides*; *Trichuris trichiura*; *Schistosoma haematobium*; parasite control programme; water contact.

## Introduction

The global prevalence of urogenital schistosomiasis (*Schistosoma haematobium*) is estimated to be about 200 million with 500–600 million people from 74 developing countries at risk of being infected because of inadequate sanitary facilities. This is as a result of poverty, poor housing and sub-standard hygiene.^1^ Approximately 80% of those infected with *S. haematobium* and *Schistosoma mansoni* are in sub-Saharan Africa.^2^

An estimated 4.5 billion people are infected with one of the three common soil-transmitted helminth (STH) species, namely the roundworm (*Ascaris lumbricoides*), the whipworm (*Trichuris trichiura*) and the hookworms (*Ancylostoma duodenale* and *Necator americanus*).^3^ It is reported that globally, 1221 million people are infected with *A. lumbricoides*, 795 million with *T. trichiura* (whipworm) and 740 million people with hookworms.^4^ Children are the most affected, and most of them live in poor or malnourished populations, which contributes to the increase in morbidity and mortality.^5^ Hong et al. further noted that inadequate hygiene, poor healthcare systems and facilities, social instability, civil war and natural disasters further exacerbate the situation.^5^

In general, the growth and survival of schistosome parasites and their snail intermediate hosts are sensitive to changes in weather patterns.^6^ Previous studies have revealed that there is evidence that *S. mansoni* in South Africa extends from Musina in Limpopo down to Port St. Johns and Port Elizabeth in the Eastern Cape.^7^ Twenty years ago, infections (> 70%) were reported in Mpumalanga.^7^ Other rural areas of KwaZulu-Natal such as Umbumbulu and Mtunzini had a prevalence of 51% – 70%, and lower prevalence of schistosomiasis presented in the coastal regions of the province including Port Shepstone, which is the site of the study reported in this article.^8^

In 1938 and confirmed in the 1980s, the distribution of *S. haematobium* was broader than that observed for *S. mansoni* with the highest prevalence of 70% – 100% observed in the Northern Province, Gauteng, North West Province, Mpumalanga and KwaZulu-Natal.^9,10^

Humid, warm and moist temperatures in these regions^11^ enable fast conversion of freshly deposited infertile eggs into fertile infective eggs (*A. lumbricoides* and *T. trichiura*) or infective larvae (hookworm species).

## Materials and methods

### Study population and area

A cross-sectional study was conducted in the Ugu district on the south coast of the province of KwaZulu-Natal, South Africa, between January and November 2010. Details about the study area are discussed elsewhere.^12,13,14^

### Study design and sample

Schools were randomly selected to participate in the study based on the distance from the coast (> 10 km), using the altitude map to locate potential schools situated below 300 m because schistosomiasis prevalence decreases with an increase in altitude.^15^ Eighteen schools were selected, and 17 of these schools were classified by the Department of Education as rural and one school as urban. The study population included female pupils aged 10–12 years old. All girls in this age range were invited to participate. Parents were informed about the study and its objectives at the schools’ parents’ meetings. Informed consent and assent to participate in the study were obtained from the parents and pupil, respectively. Of 1948 eligible girls from 18 randomly selected primary schools, 1241 (64%) consented to participate in the study, of whom 1057 participated and were interviewed. Although parental consent was provided, 15% (*n* = 184) of children did not attend school on the days that the interviews were conducted and the samples were collected. The mean age was 11.1 (standard deviation [s.d.] 0.85) years. The age of two pupils was not included.

### Parasitological assessment

Urine samples were collected per participant on three consecutive days, requiring each school to be visited on three occasions. Clean pre-labelled ‘honey’ jars with unique identity numbers were used to collect urines between 10:00 and 14:00 h each day. One stool sample was collected per participant in a pre-labelled specimen jar. The collected samples were packed in cooling boxes with ice packs to maintain temperatures around 4 °C. They were transported to the laboratory that had been set up in the field for processing by trained research assistants on the same day. *Schistosoma haematobium* infections were determined by egg count microscopy using the urine centrifugation method and categorised as per the World Health Organization (WHO) quantification guidelines as mild (1–49 egg per 10 mL urine) or heavy (≥ 50 eggs per 10 mL urine).^16^ Individual stool samples were processed in duplicate according to the Kato–Katz technique on the following day. Slides were examined by trained research assistants for the presence of ova of *S. mansoni, T. trichiura, A. lumbricoides* and hookworm species. The egg count was performed and the intensity of infection was classified according to the WHO quantification guidelines.^16^

### Data analysis

The prevalence and intensity of the helminth infections were analysed using univariate analysis. All data were entered and analysed using the Statistical Programme for Social Sciences version 18 (SPSS 18) statistical package. A χ^2^ test was used to analyse associations between the prevalence of schistosomiasis and factors such as knowledge about schistosomiasis and reporting red urine, and between the different helminth infections. The value *p* < 0.05 was considered significant and 95% confidence intervals (CI) are provided.

### Ethical considerations

This study was given ethical clearance by the University of KwaZulu-Natal’s Biomedical Research Ethics Committee (Ref: BF005/12). The Departments of Health and Education in Ugu district, KwaZulu-Natal, also gave permission for this study. All subjects found positive for urinary and intestinal schistosomiasis were treated with single dose (40 mg/kg) praziquantel, and those infected with soil-transmitted helminths were referred to primary health clinics (PHC) for treatment, because PHC clinics held stocks of the latter.

## Results

### Prevalence and intensity of helminth infections

Female pupils (*N* = 970) aged 10–12 years provided urine samples and 853 pupils provided stool samples. Table 1 shows the prevalence and mean intensities of *S. haematobium* if less than three urine samples were collected.

**TABLE 1:** Prevalence and intensity of *Schistosoma haematobium* per number of urine samples collected per study subject.

Number of urine samples collected	*S. haematobium* (positive)		Mean intensity eggs per 10 mL (s.d.) *S. haematobium*	
	*n*	%	s.d.	Mean intensity
Pupils (*n* = 29) providing one urine sample	7	24.1	44	10.6
Pupils (*n* = 150) providing two urine samples	36	24.0	60	18.3
Pupils (*n* = 791) providing three urine samples	269	34.0	32	9.6
Total urine samples (*n* = 970)	312	32.2	57	16.7

s.d., standard deviation.

The prevalence of pupils found to be infected with *S. haematobium* was 32.2% (95% CI 30.1% – 37.4%) (Table 1). Schistosomiasis was found in all the schools with a prevalence as high as 66.7% in some schools. One-third of the investigated schools was found to have schistosomiasis prevalence above 40.0% ranging from 40.0% to 66.7% (Table 2).

**TABLE 2:** Prevalence of Schistosoma haematobium, Ascaris lumbricoides and Trichuris trichiura amongst pupils (aged 10–12 years) attending 18 rural KwaZulu-Natal primary schools.

Pupils count per school		Prevalence (%) of S. haematobium (*n* = 1035)	95% CI	Prevalence (%) of A. lumbricoides (*n* = 853)	95% CI	Prevalence (%) of T. trichiura (*n* = 853)	95% CI
School	*n*						
A	100	43	33–56	35	25–45	42	32–52
B	71	40	25–56	32	14–33	28	1–30
C	37	50	27–73	40	22–61	25	6–28
D	114	38	26–52	26	17–38	48	36–61
E	11	20	3.6–62	11	2–43	22	6–55
F	50	20	11–35	25	15–39	27	17–41
G	37	36	20–55	19	9–35	19	9–39
H	101	47	36–57	24	16–35	37	27–47
I	44	48	33–62	37	24–52	29	18–44
J	46	54	39–68	38	25–52	47	33–61
K	172	11	7–16	19	14–26	16	12–23
L	77	17	9–29	19	11–30	21	13–32
M	63	8	2.6–20	27	17–40	14	7–24
N†	34	13	5–29	20	10–37	17	14–44
O†	22	10	3–30	15	5–37	5	1–24
P†	7	25	5–70	16	3–56	0	0–4
Q†	42	9	3–23	0	0–10	0	0–10
R†	7	67	36–92	14	3–51	0	0–35

CI, confidence interval.

† , The last five schools had few pupils as these schools were affected by the teachers’ strike (2010) and were visited during the time teachers were trying to catch up the lost time and preparing for final exams.

The mean intensity for pupils infected with *S. haematobium* was 52 eggs per 10 mL (s.d. 90.3). A total of 71.8% (224 out of 312) of infected pupils had a low intensity of infection. There was a significant difference (*p* < 0.05) in the intensity of schistosomiasis between schools.

*Ascaris lumbricoides* and *T. trichiura* (Table 3) were found, but no hookworm species (spp.) or *Taenia* spp. Of the pupils, a proportion of 221 (25.9%) were infected with *T. trichiura* and *n* = 209 (24.5%) with *A. lumbricoides*. None of the schools had an STHs prevalence above 50.0%. Ten of the schools had a prevalence of *A. lumbricoides* and *T. trichiura* of 20.0% or above (Table 2).

**TABLE 3:** Mean intensity (eggs per gram) of *Schistosoma haematobium, Ascaris lumbricoides* and *Trichuris trichiura* amongst pupils attending 18 KwaZulu-Natal primary schools.

School ID (*n*)	*S. haematobium*		*A. lumbricoides*		*T. trichiura*	
	Eggs per 10 mL urine	± s.d.	Eggs per gram	± s.d.	Eggs per gram	± s.d.
Overall 18 schools	17	56	4110	14 841	146	641
School A (100)	19	47	4956	12 403	188	411
School B (71)	7	12	9640	26 182	139	350
School C (37)	23	38	7091	17 274	49	88
School D (114)	31	81	9689	37 928	141	449
School E (11)	1	3	1951	5853	102	246
School F (50)	47	121	2762	6142	140	554
School G (37)	11	46	2380	5928	231	1006
School H (101)	30	70	2229	5490	319	1305
School I (44)	40	98	5197	8408	41	82
School J (46)	32	66	5635	8569	253	791
School K (172)	4	16	2239	5684	116	602
School L (77)	4	11	2691	6386	85	208
School M (63)	3	12	4446	8127	133	535
School N† (34)	7	20	1726	4775	197	967
School O† (22)	0.44	1	2097	5811	17	76
School P† (7)	54	99	210	514	0	0
School Q† (42)	0.86	0.3	0	0	0	0
School R† (7)	14.8	17	2857	7559	0	0

s.d., standard deviation.

† , The last five schools had low number of pupils as these schools were affected by the teachers’ strike (2010) and were visited during the time teachers were trying to catch up the lost time and preparing for final exam.

Fifteen of the schools had pupils infected with both ascariasis and trichuriasis and two schools had pupils infected with one helminth species. There was a statistically significant association between the prevalence of *T. trichiura* and *S. haematobium* (*p* < 0.05) within the schools; however, significant differences in infections between schools were found for all the infections found in the study (*p* < 0.01).

The burden of ascariasis and schistosomiasis varied per school from those with a low mean intensity (210 eggs per gram) to moderate infection (9689 eggs per gram). For trichuriasis, the intensity of infection in all schools was low (Table 3). An intensity of infection of 146 eggs per gram of stool was found for T. trichiura, and this was the least intense infection of the three helminths in this population with only 24 (10.4%) cases found with moderate intensity of infection and one case (0.4%) that had high intensity. The rest were found to have low intensity of infection.

### Intensity of infection in pupils with dual and triple helminth infections

All dual infection cases had moderate to high intensities of infection (Table 4). All triple infection cases had high intensity of infection. Pupils with dual co-infections comprised almost 30% of the pupils in our study who had moderate to heavy infestation. High intensity (52 eggs per 10 mL urine) of *S. haematobium* infection was observed amongst 27.9% of infected pupils.

**TABLE 4:** Intensity of infection amongst dual- and triple-infected pupils from 18 Ugu district primary schools (*N* = 1044).

Infection category	Helminth	Number of infected pupils	Mean intensity	± s.d.
Dual infection	*-*	116	-	-
	*T. trichiura*	-	619 eggs per gram	1250
	*A. lumbricoides*	-	16 689 eggs per gram	19 189
Dual infection	*-*	85	-	-
	*T. trichiura*	-	441 eggs per gram	844
	*S. haematobium*	-	81 eggs per 10 mL	117
Dual infection	*-*	69	-	-
	*S. haematobium*	-	70 eggs per 10 mL	70
	*A. lumbricoides*	-	15 783 eggs per gram	16 732
Triple infection	*-*	44	-	-
	*S. haematobium*	-	90 eggs per 10 mL	117
	*A. lumbricoides*	-	17 819 eggs per gram	18 456
	*T. trichiura*	-	552 eggs per gram	905

s.d., standard deviation.

### Association of reported knowledge about schistosomiasis and history (*n* = 1019)

Table 5 shows that most pupils had heard about schistosomiasis (locally known as *isichenene*), and one in five knew that they had bilharzia in the past, but less than one of six knew about cases in their family. Having the knowledge and having had bilharzia were significantly associated with the current *S. haematobium* infection.

**TABLE 5:** Pupils’ knowledge and reported history of *Schistosoma haematobium* (bilharzia).

Question	*N*	Response	*n*	%
Know what bilharzia is*	1020	- No Yes Unsure	- 393 587 39	- 39 58 4
History of bilharzia within family**	1022	- No Yes Do not know	- 796 149 77	- 78 15 8
Ever had bilharzia**	1020	- No Yes Do not know	- 773 226 21	- 76 22 2
Ever treated for bilharzia*	237	- No Yes	- 102 129	- 45 54

* , Variable significant association with prevalence of *S. haematobium* (*p* < 0.05); **, Variable significant association with prevalence *S. haematobium* and *T. trichiura* (*p* < 0.05).

### Comparison of prevalence of helminth infections and water contact in 1998 and 2010

There had been a reduction of more than half in the prevalence of both *A. lumbricoides* and *T. trichiura* in female pupils aged 10–12 years. In addition, in 1998, the overall prevalence for both helminths, for all pupils investigated (ages 5–14 years), was more than twice what we found in our study. However, the current prevalence of *S. haematobium* was found to be significantly higher than the findings from the parasite control programme (PCP) in 1998. The water contact seems to be dramatically reduced since 1998.

The PCP study was conducted in the same area and reported a mean egg count of 22.5, s.d. 57.2 eggs per 10 mL urine for *S. haematobium*, which was similar to that found in our study 16.7, s.d. 56.4 eggs per 10 mL urine (Table 6).

**TABLE 6:** Comparison of prevalence and intensity of helminth infections and water contact in 1998 and 2010 studies in Ugu district.^20^

Infection and water contact status	1998 (*n* = 398)						2010 (*n* = 1057)					
	%	95% CI	Eggs per 10 mL urine		Eggs per g stool		%	95% CI	Eggs per 10 mL urine		Eggs per g stool	
			*n*	s.d.	*n*	s.d.			*n*	s.d.	*n*	s.d.
**Prevalence**												
*S. haematobium*	15	12–19	-	-	-	-	32.2	29–35	-	-	-	-
*A. lumbricoides*	62	58–67	-	-	-	-	24.5	22–27	-	-	-	-
*T. trichiura*	59	54–63	-	-	-	-	25.9	23–29	-	-	-	-
**Intensity**												
*S. haematobium*	-	-	22.5	57	-	-	-	-	16.7	56	-	-
*A. lumbricoides*	-	-	-	-	73	64	-	-	-	-	4110	14 841
*T. trichiura*	-	-	-	-	65	60	-	-	-	-	146	642
**Water contact**												
Fetch water from river	72	67–76	-	-	-	-	8.0	6–10	-	-	-	-
Wash clothes in river	44	39–49	-	-	-	-	12.0	10–15		-	-	-
Swim in the river	48	43–53	-	-	-	-	0.5	0.2–1	-	-	-	-
Play in the river	47	43–53	-	-	-	-	0.5	0.2–1	-	-	-	-
Other water contact†	11	8–15	-	-	-	-	63.0	60–66	-	-	-	-

Note: Data from 1998 is from unpublished findings from the Parasite Control Program.

s.d., standard deviation; CI, confidence interval.

† , Unlisted freshwater contact.

## Discussion

A third of pupils investigated tested positive for *S. haematobium* infection, a quarter of pupils investigated tested positive for *T. trichiura* and almost a quarter of pupils investigated were infected with *A. lumbricoides*. Ten per cent were co-infected with both STHs. No other STHs were found in the study. The majority had light and moderate intensities of infection. The PCP found three species of STHs, whereas in our study, we only identified two species as no hookworms were found in our study. Hookworm species ova hatch fast and the method used in our study to analyse stool samples (Kato–Katz method) is not optimal to detect hookworm species; also the delay in analysing stool samples (next day) may have further reduced the possibility of finding hookworm species ova.^17^ The prevalence of *A. lumbricoides* and *T. trichiura* had decreased by more than half, but the prevalence of *S. haematobium* had increased when compared with the 1998 PCP study.

The high variability of multiple *S. haematobium* counts makes single urine sample less accurate for estimating total prevalence and for identifying the infection status of individuals.^18,19^ The schistosomiasis prevalence amongst the 791 pupils who gave one urine was lower than the overall prevalence for all urines collected. Only 853 of the participants in the study gave stool samples and about a quarter of these were infected with STHs found in our study.

There has been a decrease in the prevalence of STH infections since the PCP study, which was initiated in 1998 and concluded in the year 2000. *Ascaris lumbricoides* and *T. trichiura* were reported to be 24.5% and 25.9%, respectively, in 2010, compared to 62.4% and 58.6% in 1998, whereas *S. haematobium* had increased from 14.9% in 1998 to 32.2% in 2010.^20^ Temperature, dryness and ultraviolet light are the main factors that can influence the death of helminth eggs.^21^ Ugu district has not faced drought in recent years, and there have been efforts to improve water and sanitation facilities at household level which may have contributed towards a reduction in the prevalence of helminth infections.^22^

South Africa has a history of endemicity of STH infections.^9,23^ A prevalence of *A. lumbricoides* of 70% was noted amongst children in Cape Town attending the outpatients’ department of the Red Cross War Memorial Children’s Hospital.^23^ A survey of inpatients at King Edward VIII Hospital in Durban yielded 80% positive for helminths,^24^ whilst a survey amongst Cape school children in the Tygerberg area reported a prevalence of 96%.^25^ The prevalence found in Ugu district in 2010 was much lower.

Studies conducted in other parts of the province of KwaZulu-Natal in the past have reported prevalence similar or higher to those in this study. In a study that investigated the prevalence of ascariasis and other helminths in children attending a rural KwaZulu-Natal hospital and its referring clinic, 38.0% and 22.0% were found to be positive for *A. lumbricoides* and *T. trichiura*, respectively.^26^ A study conducted in northern KwaZulu-Natal school pupils found a prevalence of *S. haematobium* infection of 68.0%.^19^ A proportion of 59.8% and 22.0% pupils were found to be infected with *T. trichiura* and *A. lumbricoides*, respectively.^27^ Whilst these findings from the past studies show us that the problem of helminths has been present for some time in the province of KwaZulu-Natal, they also indicate that *S. haematobium, A. lumbricoides* and *T. trichiura* are co-endemic in many regions of the province.

These health problems can be reversed by administering the recommended treatment for STHs (mebendazole or albendazole) and for schistosomiasis (praziquantel) using the correct dose (40 mg/kg). However, if untreated, the damage caused by urogenital schistosomiasis cannot be reversed even though egg deposition can be reduced significantly. Urogenital schistosomiasis can result in bladder, ureter and kidney diseases, which are often diagnosed at the later stages of infection. Bladder cancer is another possible late-stage complication. In women, urogenital schistosomiasis may lead to genital lesions, vaginal bleeding, pain during sexual intercourse and infertility.^28,29^ Male pupils also suffer immensely from infection if they are not treated. Urogenital schistosomiasis infections can induce pathology of seminal vesicles, prostate and of other organs and may have long-term irreversible effects, including infertility.^30^

In coastal Kenya, age-stratified analysis (< 12 years) showed that overall children in the older (12–20 years) age groups had less infection than those in the younger (5–11 years) age groups, although at the outset of the programme in 1984, the older children had the higher prevalence (71%) compared with the younger age groups (63%).^31^ Older children may have a lower risk for infection because of an acquired immunity that is not found in younger children, as was suggested by earlier studies on immune response and reinfection.^32,33,34,35,36,37^ Satayathum et al. in the findings published in 2006 showed that gender-specific prevalence of infection pattern over years was not constant as they found over a period of 9 years of study, indicating that a number of factors may influence gender-specific prevalence.^31^

The majority of cases found in our study were light infections. These findings are in agreement with the findings from the PCP study that reported overall light and moderate intensity of infections.^20^ Co-infections are common in endemic areas, and Bradley and Buch also reported cases of *Ascaris, Trichuris* and *Taenia* co-infections in earlier studies conducted in the province.^26^

Soil transmitted helminths eggs can survive 10–12 months in the soil upon excretion in tropical climate conditions^2,38,39^ with *Ascaris* eggs being the most resistant.^40^ This characteristic of helminths ova may enable an increase in co-endemicity through introduction of new infections, whilst old infections have not yet been eradicated. Amongst all the 18 schools included in the study, at least two of the three targeted helminths were found in pupils. These findings confirm that these areas are endemic for all three targeted helminths and highlight the importance of improved sanitation, access to clean water and helminth treatment to reduce the prevalence and intensity of these infections in children. This study targeted only female pupils but male pupils are also likely to be equally infected.

Only female pupils aged 10–12 years were included in our study as compared to both boys and girls from ages 5–17 years. Previous studies have reported that children aged 9–13 years are most vulnerable and most likely to contact helminths infection; hence, our study focused on the most vulnerable age group making it possible to arrive to general conclusion about prevalence status of all children including boys. Also, we are able to draw conclusion for both boys and girls using evidence from findings from previous studies which show that both boys and girls are infected by helminths similarly.

Towards the end of 2010, there was massive teachers’ strike that lasted for couple of weeks towards the end of the academic year. This strike meant that the interaction we had with schools was very limited when the strike was over as teachers were making up for the lost time and also preparing for final exams. This led to last five schools we visited having low participants. This was noted in our findings.

The following recommendations were made. To achieve maximum health impact, greater focus is needed in ensuring installation of safe hygienic toilets and safe water for personal and domestic hygiene in communities.

Proper sanitation facilities should be installed at the same time as water facilities to significantly effect optimum reduction of water-faecal-related infections. Access to water supply should be as close as possible to each house to reduce the exposure to unsafe water sources and maximise hygiene practices. School and health programmes should emphasise hygiene education to encourage personal hygiene and promote early health-seeking behaviour. Pupils and communities should be engaged about infections or diseases that are endemic in their communities and collective solutions should be explored as to how such diseases can be prevented from spreading.
